# Distal radial access for neuroangiography and neurointerventions: systematic review and meta-analysis

**DOI:** 10.1186/s12883-023-03416-y

**Published:** 2023-11-15

**Authors:** Jian Wang, Lin Ma, Huaxiu Cai, Huan Zeng, Fang Pei, Jun Cao, Maogang Li, Gang Cao

**Affiliations:** 1 Department of Neurology, Ganzhou Hospital of Guangdong Provincial People’s Hospital, Ganzhou Municipal Hospital, Ganzhou, 341000 China; 2 Department of Ultrasonography, Ganzhou Hospital of Guangdong Provincial People’s Hospital, Ganzhou Municipal Hospital, Ganzhou, 341000 China; 3 Department of Cardiology, Ganzhou People’s Hospital, Ganzhou, 341000 China; 4 Department of Radiology and Imaging, Ganzhou Hospital of Guangdong Provincial People’s Hospital, Ganzhou Municipal Hospital, Ganzhou, 341000 China; 5 Department of Cardiology, Ganzhou Hospital of Guangdong Provincial People’s Hospital, Ganzhou Municipal Hospital, Ganzhou, 341000 China; 6 Department of Neurological Surgery, Ganzhou Hospital of Guangdong Provincial People’s Hospital, Ganzhou Municipal Hospital, Ganzhou, 341000 China

**Keywords:** Distal radial access, Cerebral angiography, Neuroangiography, Neurointervention

## Abstract

**Background:**

Many studies have shown that coronary angiography (CAG) and percutaneous coronary intervention (PCI) via distal radial access (DRA) are safe and effective. Safety and efficacy of neuroangiography and neurointerventions via DRA are unknown.

**Purpose:**

Search the literatures on neuroangiography and neurointerventions via DRA and conduct a systematic review and meta-analysis.

**Methods:**

PubMed, Embase and Cochrane were searched from inception to November 10, 2022. After literature screening, data extraction and assessment of literature quality, random effects model was used for meta-analysis.

**Results:**

A total of 236 literatures were retrieved, and 17 literatures including 1163 patients were finally included for meta-analysis.The pooled access success rate was 0.96 (95% confidence interval, 0.94–0.98), and the heterogeneity was obvious (I^2^ = 55.5%). The pooled access-related complications incidence rate was 0.03 (95% confidence interval, 0.02–0.05), and the heterogeneity was not obvious (I^2^ = 15.8%).

**Conclusion:**

Neuroangiography and neurointerventions via DRA may be safe and effective. DRA is an alternative access for neuroangiography and neurointerventions.

**Supplementary Information:**

The online version contains supplementary material available at 10.1186/s12883-023-03416-y.

## Introduction

Currently, neuroangiography and neurointerventions are performed via transfemoral access (TF) in most hospitals. Access-related complications associated with transfemoral access include retroperitoneal hematoma, femoral arteriovenous fistula, femoral pseudoaneurysm, and so on. They are often lethal and very difficult to manage. The incidence rates of retroperitoneal hematoma ranged between 0.03% and 5%, the incidence rates of femoral arteriovenous fistula ranged between 0% and 0.27%, and the incidence rates of femoral pseudoaneurysm ranged between 0.03% and 3.23% [1].

In recent years, many neurointerventional specialists have been exploring new approaches, including transradial access (TRA), distal radial access (DRA), and so on. Studies have shown that TRA for neuroangiography and neurointerventions is safe and effective [2–5]. There are generally no fatal access-related complications associated with TRA. TRA does not also require bed rest, recovery time is shorter, and patient satisfaction is higher. However, TRA also has some disadvantages, including radial artery occlusion (RAO), osteofascial compartment syndrome and so on. At present, there are few data on the incidence rates of RAO after neuroangiography and neurointerventions through the TRA. The incidence rate of RAO after coronary angiography (CAG) and percutaneous coronary intervention (PCI) through the TRA was 3.7% [6]. Once the osteofascial compartment syndrome occurs, it could also cause disability.

Some studies suggest that DRA may also be safe and effective [7–23]. DRA can avoid osteofascial compartment syndrome and significantly reduce the rate of RAO. At present there is a lack of relevant systematic review and meta-analyse. So we conducted this study.

## Methods

### Search strategy

We conducted this systematic review and meta-analysis in accordance with the PRISMA statement. As all the studies on neuroangiography and neurointerventions through DRA were published after 2017, PubMed, Embase and Cochrane were searched from January 1, 2017 to November 10, 2022. There are no subject headings for DRA, so we used free terms for retrieval, including snuffbox*, distal transradial*, distal radial* and dorsal radial*. The subject heading of neuroangiography in PubMed and Cochrane is “cerebral angiography”, and the subject heading of cerebral angiography in Embase is “brain angiography”. There are no subject headings for neurointervention. We used subject headings and random words to retrieve neuroangiography and neurointervention. Detailed search strategies for PubMed, Embase, and Cochrane database are presented in the Supplementary Materials section. In addition, we traced and read all references of relevant reviews, meta-analyses and 38 articles that read the full text to identify any eligible studies.

### Inclusion and exclusion criteria

Included studies must meet the following criteria: (1) the subjects were patients undergoing neuroangiography or neurointerventions such as carotid artery stenting, aneurysm treatment, stroke thrombectomy, intracranial stenting, vasospasm treatment and so on; (2) DRA was the first choice of access for neuroangiography and neurointerventions; (3) original data regarding their outcomes performing DRA is available; (4) the number of cases was more than 10. The excluded literatures were as follows: article in non-English, case report, review, meta-analysis, systematic review, letter, video, editorial, protocol, comment, meeting abstract and technical note.

### Data extraction

Two trained and experienced researchers independently screened literatures according to inclusion and excluding criteria. Two other researchers independently extracted data from included literatures according to the formulated tables. If there were any disagreements, discussed them with the corresponding author and resolved them. We also contacted the corresponding authors of the included literature for missing data.

Access success was defined as successful insertion of sheath and successful catheterization of the first vessel. Access time was defined as the time from the beginning of punctures to successful catheterization of the first vessel.

### Statistical analysis

STATA 17 software was used for the data analysis. Meta-analysis of single rates was performed using random-effects model (M-H heterogeneity test). The Cochrane Q and I^2^ tests were used to evaluate the heterogeneity among the studies, with I^2^ > 50% indicating moderate to high heterogeneity. Publication bias test and the sensitivity analysis are of little significance for the meta-analysis of single rates, so this study don’t conducted them.

## Results

### Study selection process

**Fig. 1:**
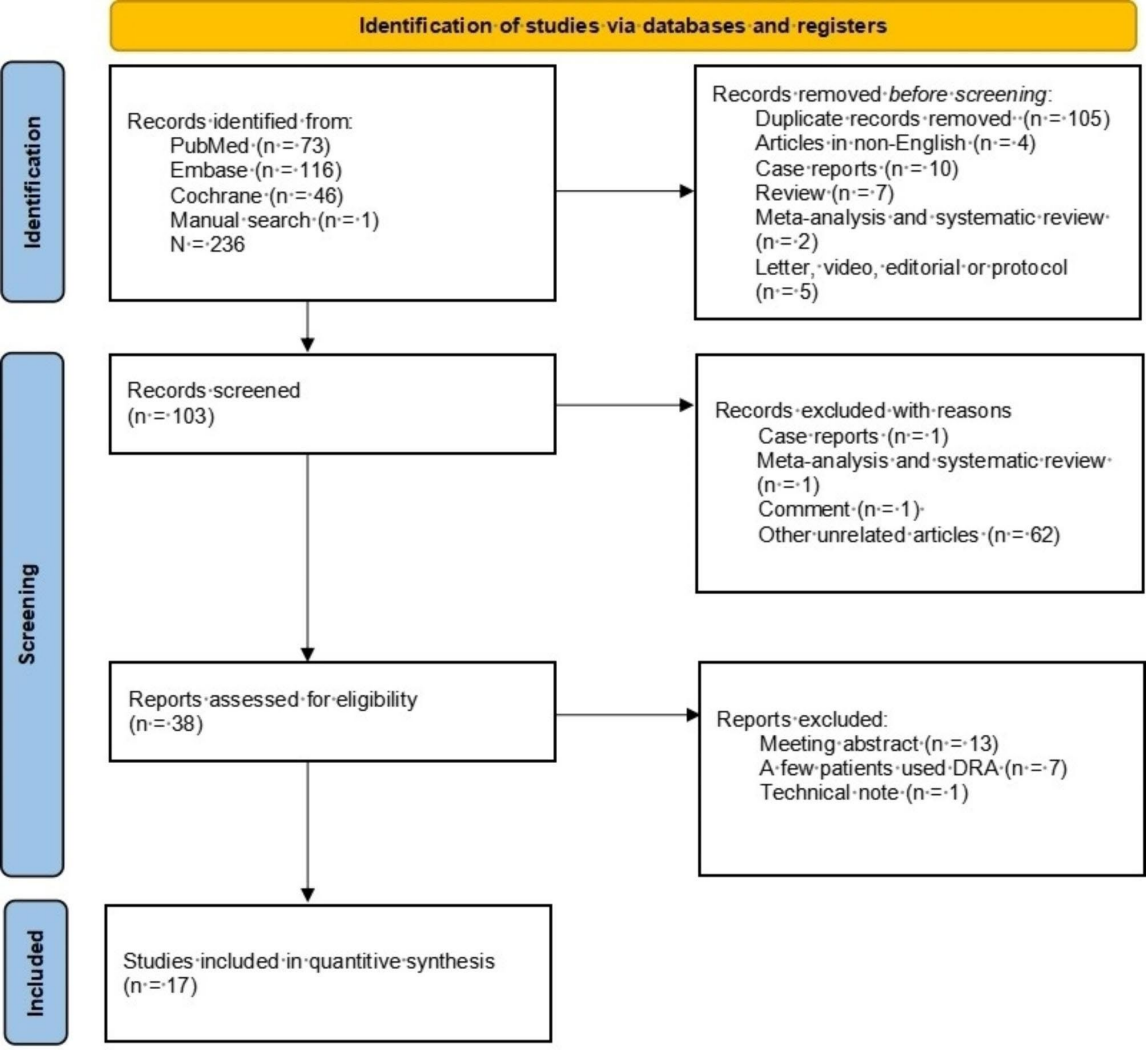
Study selection process

A total of 236 literatures were retrieved, including 73 from PubMed, 116 from Embase, 46 from Cochrane, and 1 from manual search. These literatures were imported into NoteExpress literature management software. Firstly, 105 duplicate records were removed. Secondly, 4 articles in non-English, 10 case reports, 7 reviews, 2 meta-analyses were excluded. Thirdly, a total of 5 letters, video, editorial or protocol articles were excluded. After the titles and abstracts of the remaining 103 literatures were read, 65 of them were excluded. After full texts of the remaining 38 literatures were read, 21 of them were excluded. Finally, 17 literatures were included for systematic review and meta-analysis [7–23]. The literature screening process was shown in Fig. 1.

### Quality evaluation of included studies

The quality of the included studies was evaluated according to the Newcastle Ottawa Scale modified for case series [24], Table 1. Seven studies did not report whether cases were consecutive [9, 10, 15, 19–21, 23]. Among the 17 included studies, 13 studies reported follow-up during hospitalization, 3 studies reported follow-up after discharge, and 1 study reported no follow-up.

**Table 1:** Quality evaluation of included studies

study	Selection	Ascertainment		Causality		Reporting
	Do the patients represent the whole experience of the investigator?^a^	Was the exposure adequately ascertained?	Was the outcome adequately ascertained?^b^	Were other alternative causes that may explain the observation ruled out?	Was follow-up long enough for outcomes to occur?^b^	Are the cases described with sufficient detail?
Brunet et al. [7]	Yes	Yes	Yes	Yes	No	No
Chalouhi et al. [8]	Yes	Yes	Yes	Yes	No	Yes
Patel et al. [9]	NR	Yes	Yes	Yes	No	Yes
Chivot et al. [10]	NR	Yes	Yes	Yes	No	Yes
Hoffman et al. [11]	Yes	Yes	Yes	Yes	No	Yes
Saito et al. [12]	Yes	Yes	Yes	Yes	Yes	Yes
Hoffman et al. [13]	Yes	Yes	Yes	Yes	No	Yes
Kühn et al. [14]	Yes	Yes	Yes	Yes	No	Yes
Rodriguez et al. [15]	NR	Yes	Yes	Yes	Yes	Yes
Weinberg et al. [16]	Yes	Yes	Yes	Yes	No	Yes
Manzoor et al. [17]	Yes	Yes	Yes	Yes	No	Yes
Umekawa et al. [18]	Yes	No	Yes	No	No	Yes
Goland et al. [19]	NR	NR	Yes	No	NR	No
Ahmed et al. [20]	NR	Yes	Yes	Yes	No	Yes
Kühn et al. [21]	NR	Yes	Yes	Yes	No	Yes
Chivot et al. [22]	Yes	Yes	Yes	Yes	Yes	Yes
Kühn et al. [23]	NR	Yes	Yes	Yes	No	Yes

NR: not reported

^a^ This criterion was met if authors reported consecutive series of patients

^b^ Follow-up was considered sufficient if authors reported any delayed follow-up after the procedure in the form of telephone interviews, clinical examinations, or sonography evaluations of the distal radial artery

### Characteristics of the included studies

Characteristics of the included 17 studies were shown in Tables 2 and 3. The studies were published between 2019 and 2022, and 10 studies were conducted in the United States. There were 1163 cases in all, the average or median age is 41.9 to 69.4 years old. The proportion of male ranged from 18.2 to 85%. Two studies did not report the access was the right DRA or the left DRA. Ten studies reported the mean distal radial artery diameters ranging from 2 to 2.4 mm. Seven studies included only patients with neuroangiography, four studies included only patients with neurointerventions, and the other six studies included both patients with neuroangiography and patients with neurointerventions. Patients who failed to use the DRA were switched to the TRA or TF. Reasons for the access crossover include puncture failure, arterial spasm and anatomical variation and tortuosity of the aortic arch, carotid artery, subclavian artery, and vertebral artery. Sheath sizes ranged from 4 to 8 F. Most sheaths for neuroangiography were 5 F, and most sheaths for neurointerventions were 6 F. The most frequently used catheter for neurography was 4-5 F Simmons 2. The most frequently used catheters for neurointerventions were 6-7 F Benchmark and Fubuki. Only 2 studies reported access time, 14.22 and 17 min respectively [11, 13]. Only two studies did not use ultrasound guidance to perform distal radial artery puncture [16, 19]. Eleven studies used patent hemostasis, two studies did not used patent hemostasis [12, 18], and four studies did not report hemostasis methods [16, 17, 19, 20].

**Table 2:** Characteristics of the included studies

Study	Year	Country	No.	Age (years)	Male (%)	Right/left	Distal radial size(mm)	Procedure type	Access success(%)
Brunet et al. [7]	2019	USA	85	53.8 ± 15	21.2	NR	2.4 ± 0.6	neuroangiography	88.2
Chalouhi et al. [8]	2021	USA	20	56.7 ± 12.9	25	left	NR	neuroangiography	90
Patel et al. [9]	2019	USA	34	54.5 ± 11.5	50	right	≥ 2	neuroangiography	88.2
Chivot et al. [10]	2021	France	80	51(21 − 73)	47.5	left	2.1 ± 0.34	neuroangiography	98.7
Hoffman et al. [11]	2022	USA	154	56 ± 15	39	right	NR	neuroangiography	98.7
Saito et al. [12]	2020	Japan	51	59.4 ± 13.5	31.4	right 92.2%, left 7.8%	2.19 ± 0.41	neuroangiography	92.2
Hoffman et al. [13]	2021	USA	75	56.1 ± 14.8	38.7	right	NR	neuroangiography	98.7
Kühn et al. [14]	2020	USA	48	64.4(35–84)	41.3	Right 97.9%, left 2.1%	2.1(1.6–3.0)	neurointervention	89.6
Rodriguez et al. [15]	2022	Spain	100	58 ± 15.6	58	right 86%, left 12%, both 2%	2.03 ± 0.38	neuroangiography 53%,neurointervention 47%	96
Weinberg et al. [16]	2020	USA	120	54.7 ± 14.7	44.2	NR	NR	neuroangiography 92.5%,neurointervention 7.5%	100
Manzoor et al. [17]	2021	Saudi Arabia	114	41.9 ± 15.2	64.7	right,left	2.4(1.6–2.9)	neuroangiography 63.2%,neurointervention 31.6%	94.7
Umekawa et al. [18]	2022	Japan	30	67(25–87)	70	right,left	2.3(1.7–3.2)	neuroangiography 70%,neurointervention 30%	77
Goland et al. [19]	2019	Argentina,España	94	52	40.4	right,left	NR	neuroangiography 71.3%,neurointervention 28.7%	20.2
Ahmed et al. [20]	2021	USA	64	56(16–81)	50	right 93.7%, left 6.3%	NR	neuroangiography 73.4%,neurointervention 26.6%	96.9
Kühn et al. [21]	2021	USA	22	69.4(53–87)	85	right	2.1(1.6–2.8)	carotid artery stenting	90.9
Chivot et al. [22]	2022	France	61	53.5	63.9	right 85.2%, left 14.8%	2.05	cerebral aneurysm embolization	98.4
Kühn et al. [23]	2020	USA	11	63.5	18.2	right	NR	cerebral aneurysm embolization	90.9

No.:number of cases; NR: not reported

**Table 3:** Procedural characteristics and access-related complications

Study	Crossover and reason(%)	Sheath size	Catheter type	Access-related complications(%)
Brunet et al. [7]	TRA 1.2%, TF 10.6%, artery spasm,inability to cannulate the artery,arteria lusoria configuration	5 F Glidesheath Slidesheath	NR	0
Chalouhi et al. [8]	TRA 10%	5 F Prelude sheath	5 F Simmons 2	0
Patel et al. [9]	TRA 5.9%, TF 5.9%, radial artery vasospasm	5 F Prelude Ideas, 5 F Glidesheath Slender	5 F Simmons 2	wrist pain 5.9%
Chivot et al. [10]	TF 1.3%, artery spasm	5 F radial sheath	4 F vertebral catheters supported by 5 F Extra Back Up	distal RAO 1.3%, minor forearm blanching 1.3%
Hoffman et al. [11]	TF 1.3%, radial artery vasospasm, inability to cannulate the radial artery and brachiocephalic artery tortuosity	5 F Glidesheath Slender	Simmons 2, angled glide	minor hematomas,vasospasm, radial artery perforation, pain (total 5.2%)
Saito et al. [12]	TRA 7.8%, NR	4 F Slit Super-Sheath	4 F Simmons 2,4 F JB2	minor hematomas 11.8%, numbness 2%
Hoffman et al. [13]	NR 1.3%, NR	5 F Glidesheath Slender	Simmons 2, angled glide	minor hematomas 1.3%, vasospasm 1.3%, radial artery perforation 1.3%
Kühn et al. [14]	TF 10.4%, tortuous aortic arch tortuous	6 F Prelude Ideal hydrophilic sheath	Benchmark 60.5%, Fubuki 18.6%, others 20.9%	radial artery vasospasm 4.2%, distal and RAO 2.1%
Rodriguez et al. [15]	TRA 1%, TF 3%, puncture failure, arterial loop at the elbow, catheterize difficulty	5-6 F thinner wall sheath,6 F Ballast,Cook Shuttle,Neuron MAX	sheathless technique	minor hematomas 3%, distal RAO 1%, radial stenosis 2%
Weinberg et al. [16]	0	5 F 94.2%,6 F 5.8%	NR	minor wrist hematoma 0.8%, radial artery vasospasm 0.8%
Manzoor et al. [17]	TRA 2.6%, TF 1.8%, TUA 0.9%, puncture failure, artery vasospasm	4-6 F Glidesheath Slender	6 F Benchmark,sheathless technique	minor hematomas 2.6%
Umekawa et al. [18]	TRA 23%, NR	4 F Medikit sheath,6 F FUBUKI,8 F Optimo	4 F MS2, Simmons C supported by 6 F FUBUKI and 8 F Optimo	minor hematomas 3.3%
Goland et al. [19]	TRA 52.1%, TF 28.7%, NR	6 F sheath	Simmons supported by Chaperon,Guider SofTip XF 6 F,Navien,Sophia	NR
Ahmed et al. [20]	TF 3.1%, near occlusion of radial artery, right brachiocephalic artery tortuosity, aortic arch variable configuration	NR	NR	radial artery injury 1.6%
Kühn et al. [21]	TF 9.1%, radial artery vasospasm, tortuous vessel anatomy and type 3 aortic arch	6 F Prelude Ideal hydrophilic sheath 77.3%, 7 F sheath 4.5%, sheathless 18.2%	Benchmark, 5-7 F Fubuki,Select Flex 072	0
Chivot et al. [22]	TF 1.6%, humeral artery spasm	6 F sheath	NR	0
Kühn et al. [23]	TF 9.1%, brachial artery fibromuscular dysplasia	6 F Prelude Ideal hydrophilic sheath,sheathless	5 F Sofia supported by 6 F Benchmark,6 F Fubuki sheathless technique	0

### Access success rates and access-related complications

**Fig. 2:**
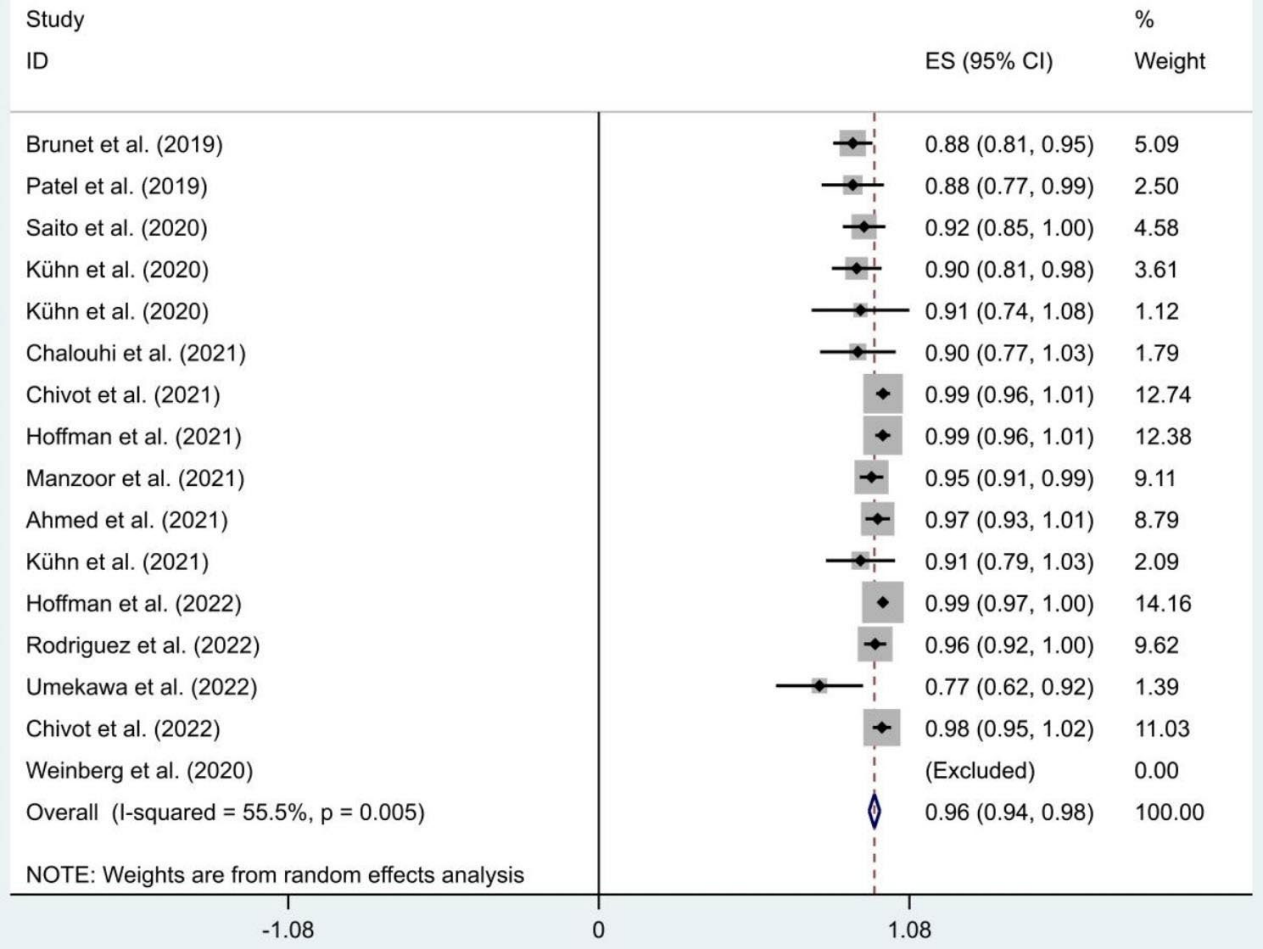
Meta-analysis of access success rate

All 17 studies reported access success rates ranging from 20.2 to 100%. Goland et al.’s study had a very low access success rate [19], which we considered to be an outlier, so we did not include it in the meta-analysis. The meta-analysis result of access success rates of 16 studies was shown in Fig. 2. The pooled effect size (ES) and 95% confidence interval (CI) was 0.96 (0.94, 0.98). The heterogeneity was obvious (I^2^ = 55.5%, P = 0.005).

**Fig. 3:**
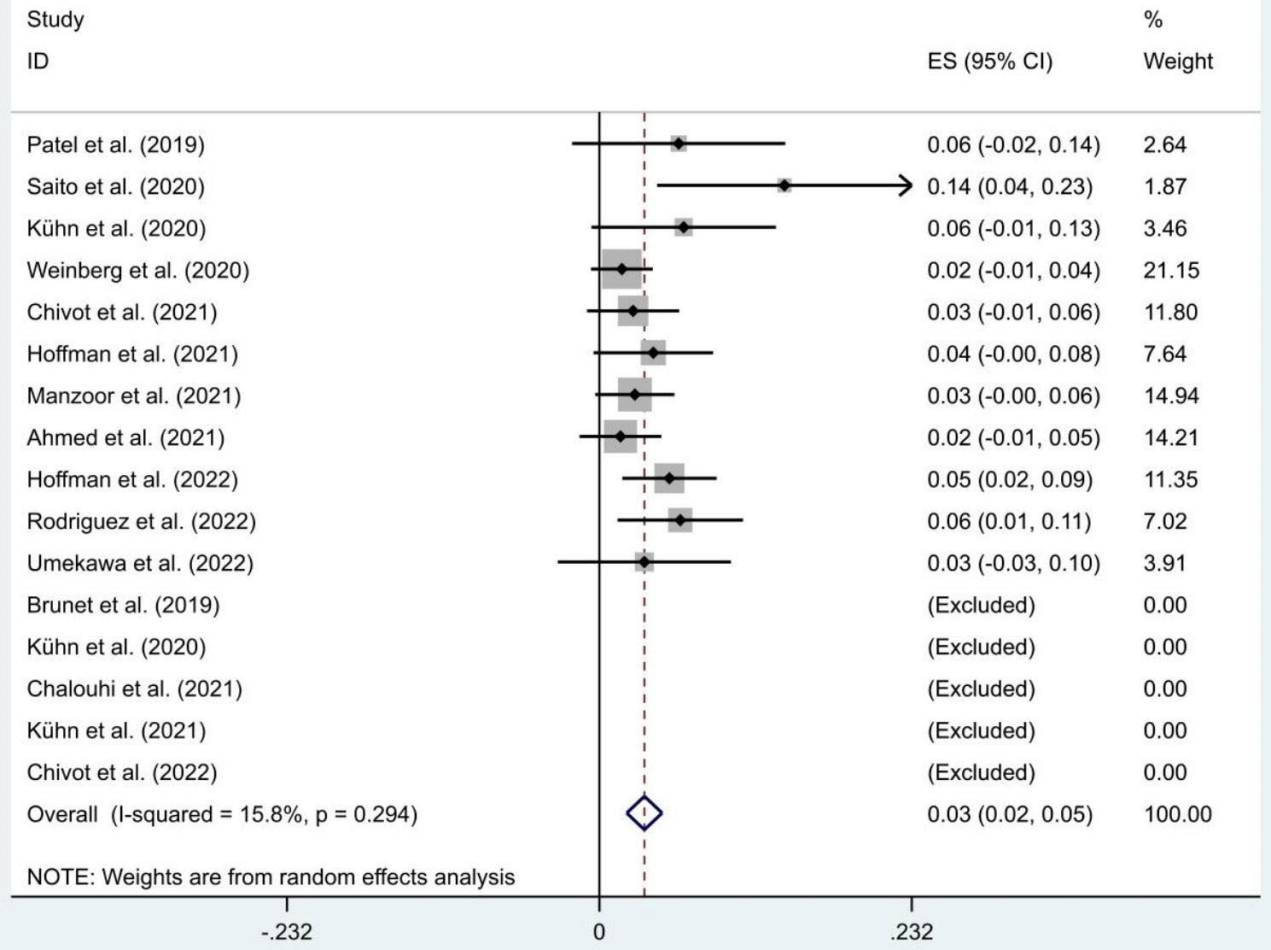
Meta-analysis of access-related complications

Goland et al.’s study did not report the incidence rate of access-related complications [19]. Access-related complications incidence rates of other 16 studies ranged between 0% and 13.7%. It included minor hematomas (0–11.8%), vasospasm (0–4.2%), distal RAO (0–2.1%), RAO (0–2.1%), numbness (0–2%), pain (0–5.9%), minor forearm blanching (0–1.3%), radial artery perforation (0–1.3%), radial stenosis (0–2%), radial artery injury (0–1.6%). Both distal RAO and RAO were asymptomatic. The meta-analysis result of access-related complications was shown in Fig. 3. The pooled effect size (ES) and 95% confidence interval (CI) was 0.03 (0.02, 0.05). The heterogeneity was not obvious (I^2^ = 15.8%, P = 0.294).

Publication bias test is of little significance for the meta-analysis of single rates, so this study don’t conducted it.

## Discussion

TRA has been the first choice for CAG and PCI for many years [25]. Due to the disadvantages of TRA such as RAO, Kiemeneij et al. [26] first reported the use of DRA for CAG and PCI in 2017. Since then, numerous studies have shown that CAG and PCI via DRA are safe and effective [27–30]. The cerebral artery is more tortuous than the coronary artery, and its operation is more complex. How about the safety and efficacy of neuroangiography and neurointerventions via DRA? We searched the relevant literature and conducted a systematic review and meta-analysis. The results of this study showed that the access success rate was high, and the incidence rate of the access-related complications was low. No serious complications were observed.

### Access success rate

In this study, access success rates varied widely, ranging from 20.2 to 100%.

The pooled access success rate was 96%, and the heterogeneity was obvious (I^2^ = 55.5%). The reason might be that many operators were beginners of DRA and included studies included a large number of cases in learning stage. In order to describe the initial experience with DRA, Goland et al. [19] specially selected the data of the first 3 months for analysis. Umekawa et al. [18] selected the first 30 consecutive cases in learning stage for analysis. Hoffman et al. [13] analyzed the first 75 cerebral angiograms performed with DRA by a single operator. Brunet et al. [7] found that in the first and second quarters of experience, 14.3% of cases were converted to traditional TRA or TF; however, in the third quarter failure rate decreased to 4.7% and 0% in the last (fourth) quarter. Therefore, with the popularization of DRA and the accumulation of experience of the operator, access success rate would be higher. However, we must acknowledge that not all patients are suitable for DRA. In some patients, the distal radial artery diameter is too small to be successfully inserted. Rodriguez et al. ‘s study excluded patients with distal radial artery diameter less than 1.7mm [15], and Manzoor et al. ‘s study excluded patients with distal radial artery diameter less than 1.6mm [17]. Both two studies had high access success rate (90.9% and 90.9%, respectively). In Tsigkas et al. ’s study [28], although all operators were experienced, the distal radial artery diameter was not measured before CAG and PCI, and patients with small distal radial artery diameter were not excluded. The access success rate of DRA was significantly lower than that of TRA (78.7% vs. 94.8%, P < 0.001). It can be concluded that excluding patients with very small distal radial artery diameter by ultrasound helps to improve the access success rate of DRA.

### Access-related complications

In this study, access-related complications incidence rates ranged between 0% and 13.7%. The pooled access-related complications incidence rate was 3%, and no serious complications were observed. According to the consensus of Korean and European cardiologists on DRA [31], no major safety issue has been reported so far among published registries. The incidence of vasospasm in this study was from 0 to 4.2% and the incidence of RAO was from 0 to 2.1%. According to the consensus of Korean and European cardiologists on DRA [31], the incidence of radial artery spasm and RAO were also very low---- only 1 RAO was registered among 1,341 patients (0.075%) enrolled in 14 observational studies. A meta-analysis of 14 studies involving 6,208 patients showed that compared with TRA, DRA was associated with a significant lower RAO risk for CAG and PCI (risk ratio [RR]: 0.36; 95% CI: 0.23–0.56; P < 0.001) [29]. The incidence of hematomas in this study was from 0 to 11.8%, which only required simple management. According to a large randomized controlled clinical trial involving 776 patients, the hematoma incidence of DRA was significantly lower than TRA in CAG and PCI [32]. Thus, compared with CAG and PCI, neuroangiography and neurointerventions via DRA is equally safe.

### Sheath and guide catheter

The diameter of femoral artery is large enough to accommodate big sheaths and guiding catheters. The diameter of radial artery and distal radial artery is smaller than femoral artery. DRA may limit the use of big sheaths and guiding catheters like 8 F sheaths and guiding catheters. Sheaths and catheters for neuroangiography in this study were from 4 to 5 F, and the diameter of distal radial artery was usually enough to accommodate them. Most sheaths and guide catheters used in neurointerventions were 6 F in this study, and a few were 7 and 8 F. The conventional 7 and 8 F sheaths and guiding catheters are too large for DRA, which is similar to PCI via DRA. In recent years, there have been a great improvement in sheaths and guiding catheters, such as sheathless guiding catheters and thin-walled sheaths. These improvements allow procedures such as carotid artery stenting and mechanical thrombectomy, which required the use of large sheaths and large guiding catheters in traditional procedures, to be performed successfully through DRA. Kühn et al. [14], Umekawa et al. [18], Kühn et al. [21], and Kühn et al. [23] all used Fubuki Neurovascular sheath (Asahi Intecc, Tokyo, Japan) in this study, which was a species of thin-walled sheaths. Rodriguez et al. [15], Manzoor et al. [17], and Kühn et al. [23] all used sheathless techniques in this study. Cao et al. ‘s study showed that the use of sheathless technique and thin-walled sheath also enabled PCI for complex coronary artery disease to be successfully performed through DRA, which required the use of large sheath and large guiding catheter in traditional procedures [33]. With the continuous improvement of operation instruments, the application of DRA in neuroangiography and neurointerventions would be more extensive.

### Limitations

Hoffman et al. [34] searched the literature on neuroangiography and neurointerventions via DRA published before August 21, 2020 in PubMed, Scopus, and Embase databases, and finally included 7 studies including 459 cases for meta-analysis. After that, some new related papers had been published. We searched the related literature published before November 10, 2022 in PubMed, Embase and Cochrane databases, and finally included 17 studies including 1163 cases for meta-analysis. Both meta-analyses showed that neuroangiography and neurointerventions via DRA were safe and effective. However, almost all the included studies were case series, and the level of evidence-based medicine was low. The sample size of the studies included in this meta-analysis was small (11 to 154), which is certainly insufficient for the incidence of RAO. Most of included studies did not perform rigorous ultrasound follow-up after procedures, which might result in patients with RAO being missed. Hemostasis methods were associated with access-related complications, and only 11 studies used patent hemostasis. Besides, studies published in non-English were not included in this study.

## Conclusions

Neuroangiography and neurointerventions via DRA maybe safe and effective. DRA is an alternative access for neuroangiography and neurointerventions. The results of this study should be considered exploratory and need to be confirmed by further prospective cohort studies and randomized controlled trials.

### Electronic supplementary material

Below is the link to the electronic supplementary material.

Supplementary Material 1

## Data Availability

All data relevant to the study are included in the article or uploaded as supplementary information.
