# Supplementary material for: Distal radial access for neuroangiography and neurointerventions: systematic review and meta-analysis
Source: BMC Neurol. 2023 Nov 15;23:405. doi: 10.1186/s12883-023-03416-y (PMC10648363; doi:10.1186/s12883-023-03416-y)
Supplement: Supplementary file 1 — Supplementary Material 1 [file 12883_2023_3416_MOESM1_ESM.docx]

**Supplementary Information**

Additional file 1: Meta-analysis of access success rate (including Goland et al. 's study)


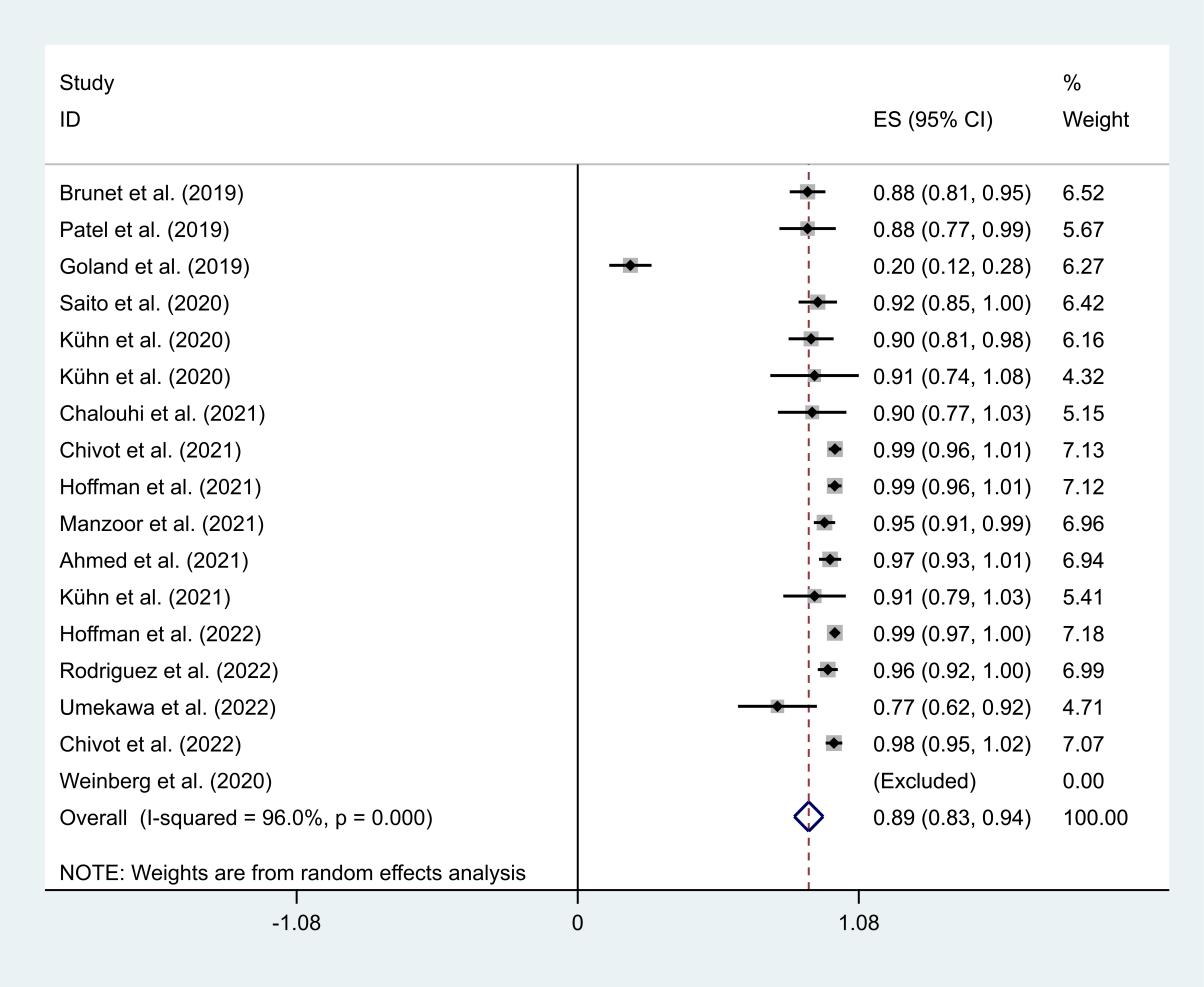


Additional file 2: Sensitivity analysis (including Goland et al. 's study)


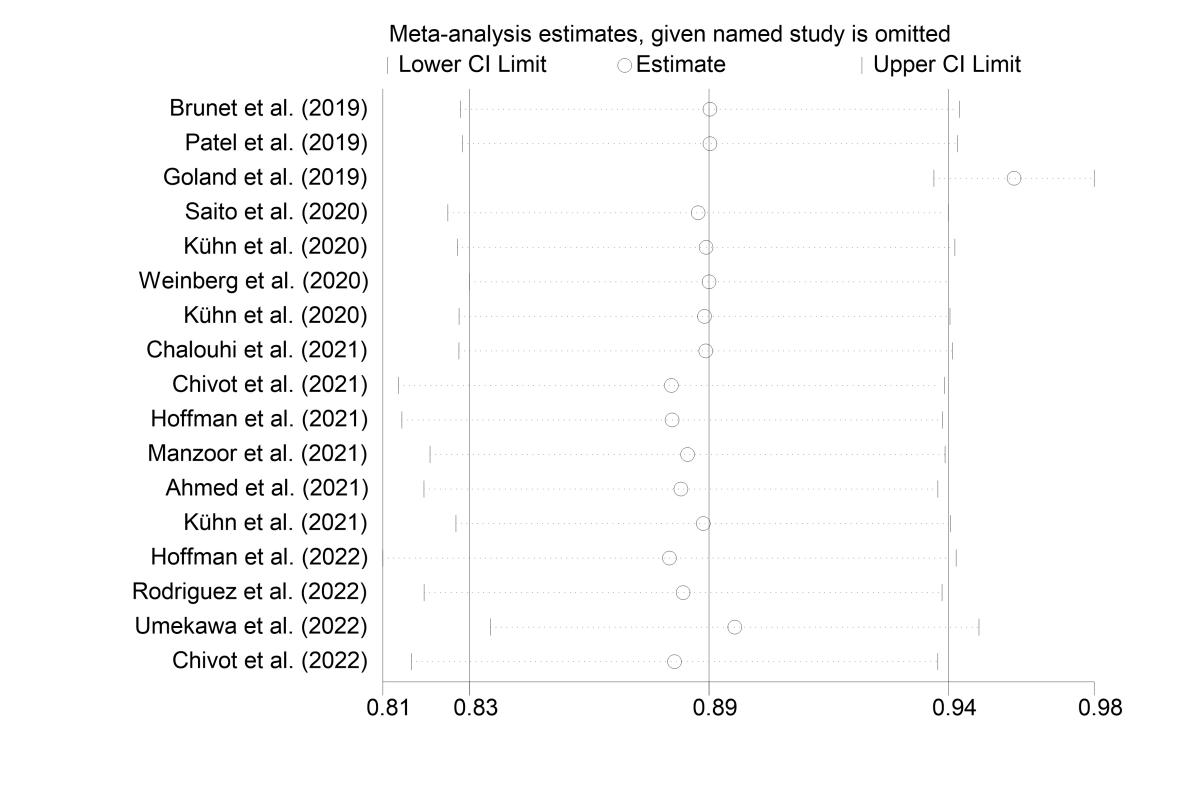


Additional file 3: Detailed search strategies

The search strategy of PubMed database was as follows: ((snuffbox*[Title/Abstract]) OR (distal transradial*[Title/Abstract]) OR (distal radial*[Title/Abstract]) OR (Dorsal Radial*[Title/Abstract])) AND (("Cerebral Angiography"[Mesh]) OR (Cerebral Angiography*) OR (Angiography, Cerebral*) OR (Angiographies, Cerebral*) OR (Cerebral Angiographies*) OR (cerebral angiogram*) OR (Neurointervention*) OR (Cerebral*) OR (Brain*) OR (Intracranial*) OR (Cranial*)). The search strategy of Embase database was as follows: ('snuffbox*':ti,ab,kw OR 'distal transradial*':ti,ab,kw OR 'distal radial*':ti,ab,kw OR 'dorsal radial*':ti,ab,kw) AND ('brain angiography'/exp OR 'brain angiography*' OR 'angiography, brain*' OR 'arteriography, brain*' OR 'brain angiogram*' OR 'brain arteriogram*' OR 'brain arteriography*' OR 'cerebral angiogram*' OR 'cerebral angiography*' OR 'cerebral arteriogram*' OR 'cerebral arteriography*' OR 'encephaloarteriography*' OR 'angiography, cerebral*' OR 'angiographies, cerebral*' OR 'cerebral angiographies*' OR 'neurointervention*' OR 'cerebral*' OR 'brain*' OR 'intracranial*' OR 'cranial*'). The search strategy in the Cochrane database was as follows: #1 = MeSH descriptor: (Cerebral Angiography) explodes all trees; #2 = (Cerebral Angiography*) OR (Angiography, Cerebral*) OR (Angiographies, Cerebral*) OR (Cerebral Angiographies*) OR (cerebral angiogram*) OR (Neurointervention*) OR (Cerebral*) OR (Brain*) OR (Intracranial*) OR (Cranial*); #3 = #1 or #2; #4 = (snuffbox*):ti,ab,kw OR (distal transradial*):ti,ab,kw OR (distal radial*):ti,ab,kw OR (Dorsal Radial*):ti,ab,kw (Word variations have been searched); and #5 = #3 and #4.

Additional file 4: Newcastle Ottawa Scale modified for case series

| Domains | Leading explanatory questions |
| --- | --- |
| Selection | 1. Does the patient(s) represent(s) the whole experience of the investigator (centre) or is the selection method unclear to the extent that other patients with similar presentation may not have been reported? |
| Ascertainment | 2. Was the exposure adequately ascertained? 3. Was the outcome adequately ascertained? |
| Causality | 4. Were other alternative causes that may explain the observation ruled out? 5. Was there a challenge/rechallenge phenomenon? 6. Was there a dose–response effect? 7. Was follow-up long enough for outcomes to occur? |
| Reporting | 8. Is the case(s) described with sufficient details to allow other investigators to replicate the research or to allow practitioners make inferences related to their own practice? |
